# High prevalence of mgrB-mediated colistin resistance among carbapenem-resistant *Klebsiella pneumoniae* is associated with biofilm formation, and can be overcome by colistin-EDTA combination therapy

**DOI:** 10.1038/s41598-022-17083-5

**Published:** 2022-07-28

**Authors:** Aye Mya Sithu Shein, Dhammika Leshan Wannigama, Paul G. Higgins, Cameron Hurst, Shuichi Abe, Parichart Hongsing, Naphat Chantaravisoot, Thammakorn Saethang, Sirirat Luk-in, Tingting Liao, Sumanee Nilgate, Ubolrat Rirerm, Naris Kueakulpattana, Sukrit Srisakul, Apichaya Aryukarn, Matchima Laowansiri, Lee Yin Hao, Manta Yonpiam, Naveen Kumar Devanga Ragupathi, Teerasit Techawiwattanaboon, Natharin Ngamwongsatit, Mohan Amarasiri, Puey Ounjai, Rosalyn Kupwiwat, Phatthranit Phattharapornjaroen, Vishnu Nayak Badavath, Asada Leelahavanichkul, Anthony Kicic, Tanittha Chatsuwan

**Affiliations:** 1 Department of Microbiology, Faculty of Medicine, Chulalongkorn University, King Chulalongkorn Memorial Hospital, Thai Red Cross Society, 1873 Rama 4 Road, Pathumwan, Bangkok, 10330 Thailand; 2 Center of Excellence in Antimicrobial Resistance and Stewardship, Faculty of Medicine, Chulalongkorn University, Bangkok, Thailand; 3 Interdisciplinary Program of Medical Microbiology, Graduate School, Chulalongkorn University, Bangkok, Thailand; 4 Department of Infectious Diseases and Infection Control, Yamagata Prefectural Central Hospital, Yamagata, Japan; 5 Biofilms and Antimicrobial Resistance Consortium of ODA Receiving Countries, The University of Sheffield, Sheffield, UK; 6 School of Medicine, Faculty of Health and Medical Sciences, The University of Western Australia, Nedlands, WA Australia; 7 Pathogen Hunter’s Research Collaborative Team, Department of Infectious Diseases and Infection Control, Yamagata Prefectural Central Hospital, Yamagata, Japan; 8 Institute for Medical Microbiology, Immunology and Hygiene, Faculty of Medicine and University Hospital Cologne, University of Cologne, Cologne, Germany; 9 German Centre for Infection Research, Partner Site Bonn-Cologne, Cologne, Germany; 10 Molly Wardaguga Research Centre, Charles Darwin University, Queensland, Australia; 11 Mae Fah Luang University Hospital, Chiang Rai, Thailand; 12 School of Integrative Medicine, Mae Fah Luang University, Chiang Rai, Thailand; 13 Department of Biochemistry, Faculty of Medicine, Chulalongkorn University, Bangkok, Thailand; 14 Center of Excellence in Systems Biology, Research Affairs, Faculty of Medicine, Chulalongkorn University, Bangkok, Thailand; 15 Department of Computer Science, Faculty of Science, Kasetsart University, Bangkok, Thailand; 16 Department of Clinical Microbiology and Applied Technology, Faculty of Medical Technology, Mahidol University, Bangkok, Thailand; 17 Department of Physiology, Faculty of Medicine, Chulalongkorn University, Bangkok, Thailand; 18 Center of Excellence for Microcirculation, Faculty of Medicine, Chulalongkorn University, Bangkok, Thailand; 19 Department of Chemical and Biological Engineering, The University of Sheffield, Sheffield, UK; 20 Department of Clinical Microbiology, Christian Medical College, Vellore, India; 21 Chula Vaccine Research Center, Faculty of Medicine, Chulalongkorn University, Bangkok, Thailand; 22 Department of Clinical Sciences and Public Health, Faculty of Veterinary Science, Mahidol University, Nakhon Pathom, Thailand; 23 Laboratory of Environmental Hygiene, Department of Health Science, School of Allied Health Sciences, Kitasato University, Kitasato, Sagamihara-Minami, Kanagawa, 252-0373 Japan; 24 Department of Biology, Faculty of Science, Mahidol University, Bangkok, Thailand; 25 Department of Dermatology. Faculty of Medicine Siriraj Hospital, Mahidol University, Bangkok, Thailand; 26 Department of Emergency Medicine, Center of Excellence, Faculty of Medicine Ramathibodi Hospital, Mahidol University, Bangkok, Thailand; 27 Institute of Clinical Sciences, Department of Surgery, Sahlgrenska Academy, Gothenburg University, 40530 Gothenburg, Sweden; 28 School of Pharmacy and Technology Management, SVKM’s Narsee Monjee Institute of Management Studies (NMIMS), Hyderabad, 509301 India; 29 Translational Research in Inflammation and Immunology Research Unit (TRIRU), Department of Microbiology, Chulalongkorn University, Bangkok, Thailand; 30 Telethon Kids Institute, University of Western Australia, Nedlands, WA 6009 Australia; 31 Centre for Cell Therapy and Regenerative Medicine, Medical School, The University of Western Australia, Nedlands, WA 6009 Australia; 32 Department of Respiratory and Sleep Medicine, Perth Children’s Hospital, Nedlands, WA 6009 Australia; 33 School of Public Health, Curtin University, Bentley, WA 6102 Australia

**Keywords:** Bacteria, Bacteriology, Biofilms, Infectious-disease diagnostics, Drug discovery, Microbiology, Medical research, Molecular medicine

## Abstract

The global prevalence of colistin-resistant *Klebsiella pneumoniae* (ColRkp) facilitated by chromosomal and plasmid-mediated Ara4N or PEtN-remodeled LPS alterations has steadily increased with increased colistin usage for treating carbapenem-resistant *K. pneumoniae* (CRkp). Our study demonstrated the rising trend of ColRkp showing extensively and pandrug-resistant characteristics among CRkp, with a prevalence of 28.5%, which was mediated by chromosomal *mgrB*, *pmrB,* or *phoQ* mutations (91.5%), and plasmid-mediated *mcr-1.1, mcr-8.1, mcr-8.2* alone or in conjunction with R256G PmrB (8.5%). Several genetic alterations in *mgrB* (85.1%) with increased expressions of Ara4N-related *phoPQ* and *pmrK* were critical for establishing colistin resistance in our isolates. In this study, we discovered the significant associations between extensively drug-resistant bacteria (XDR) and pandrug-resistant bacteria (PDR) ColRkp in terms of moderate, weak or no biofilm-producing abilities, and altered expressions of virulence factors. These ColRkp would therefore be very challenging to treat, emphasizing for innovative therapy to combat these infections. Regardless of the underlying colistin-resistant mechanisms, colistin-EDTA combination therapy in this study produced potent synergistic effects in both in vitro and in vivo murine bacteremia, with no ColRkp regrowth and improved animal survival, implying the significance of colistin-EDTA combination therapy as systemic therapy for unlocking colistin resistance in ColRkp-associated bacteremia.

## Introduction

Carbapenem resistant *Klebsiella pneumoniae* (CRkp) is frequently encountered in clinical settings^1^. Faced with resistance to all standard therapeutic options, clinicians are being urged to reconsider colistin as a viable treatment option in response to rising global CRkp prevalence, few effective therapeutic alternatives and limitations in novel antibiotic development^2,3^. Colistin, a bactericidal polycationic peptide, attaches to anionic lipid A of lipopolysaccharide (LPS), displacing Ca^++^ and Mg^++^ which form bridges between LPS, causing bacterial membrane destabilization. Colistin also triggers bacterial death through hydroxy radical-induced oxidative stress and impairment of bacterial respiratory chains^3–5^. Nevertheless, as colistin use has increased, worldwide prevalence of ColRkp has risen^3–7^.

Colistin resistance develops in *K. pneumoniae* due to a reduction in net negative charge of LPS generated by amino-4-deoxy-l-arabinose (Ara4N) and phosphoethanolamine (pEtN)-mediated LPS alterations, causing diminished electrostatic affinity between colistin and LPS^3,4^. The *pmrHFIJKLM* operon facilitates Ara4N-remodeled LPS alteration, whereas the *pmrCAB* operon supports pEtN-integrated LPS modification^3,4^. As chromosomal-mediated mechanisms, *mgrB*, *phoPQ,* and *pmrAB* mutations influence *pmrHFIJKLM* and *pmrCAB* expressions to trigger LPS alterations^3,4^. Plasmid-mediated colistin-resistant mechanisms include presence of *mcr* with different alleles (*mcr* 1–9) that encode phosphoethanolamine transferase causing PEtN-modified LPS^3,4^.

Furthermore, several studies observed establishment of hypervirulent ColRkp with diverse virulence characteristics^8–11^. In *K. pneumoniae*, biofilm development is a crucial virulence characteristic, and managing biofilms is extremely challenging as they confer significant tolerance to host defense responses and antibiotic effects^12–14^. Type 3 adhesin, *mrkD,* is implicated in bacterial adherence and biofilm development^12–14^. Hypervirulent *K. pneumoniae* displays iron-scavenging siderophores—*ybtS* and *kfu* that facilitate systemic survival by regulating immunological responses^12,15^. OmpK35 and OmpK36 are outer membrane porins that help bacteria survive by maintaining membrane integrity and delivering important nutrients^14,16^. LPS genes—*uge* and *wabG* are implicated in promoting pathogenicity by protecting bacteria against host humoral defenses^12,14,17^. Type 2 quorum-sensing regulatory system*-luxS* facilitates biofilm development by encouraging cell-to-cell communication^12,18^. It has been reported that not only PmrAB and PhoPQ support bacterial virulence by regulating virulence gene transcripts, but in addition *mgrB*-related LPS alterations also augment virulence by suppressing early host defense^19–21^, highlighting the importance of exploring the association between colistin resistance and other virulence factors that influence bacterial pathogenicity. Due to converging colistin resistance and hypervirulence, clinically untreatable *K. pneumoniae* superbugs may evolve, emphasizing the urgent need to develop viable therapeutic strategy to minimize mortality, morbidity and health-care expenses associated with these infections^8,12,22^.

Colistin combination therapy, when used to treat drug-resistant bacteria, has been shown to significantly lower treatment failure rates and enhance patient survival^23^. Ethylenediamine tetra-acetic acid (EDTA) is an anti-virulence drug that disrupts permeability-associated resistance mechanisms and restores antibiotic potency against resistant bacteria through metal ion chelation^24^, suggesting that it could be used as adjuvant in colistin combination therapy to counteract ColRkp expressing different virulence characteristics. Thus, the aims of the present study were to determine the underlying chromosomal and plasmid-mediated resistance mechanisms encoded in ColRkp, identify any association between colistin resistance and diverse virulence characteristics, and to assess the effectiveness of colistin-EDTA combination in unlocking colistin resistance in ColRkp with different colistin-resistance mechanisms.

## Results

### Prevalence of colistin resistance among CRkp clinical isolates was on the rising trend

Between 2016 and 2021, 165 CRkp retrieved from hospitalized patients exhibited different antibiotic resistance profiles, with highest resistance to ceftazidime and ciprofloxacin (100%), followed by imipenem (94.5%), meropenem (90.3%), fosfomycin (31.5%) and amikacin (23.6%). Of these, 47 isolates (28.5%) showed colistin resistance, with their incidence growing rapidly over time from 14.9% in 2016 to 36.2% in 2021 (Table 1) (Supplementary Fig. 1). The majority of ColRkp isolates (91.5%) exhibited extensively drug-resistance with the remaining 8.5% pan-drug-resistant (Table 2). Furthermore, when compared to their corresponding planktonic MIC, biofilms of these isolates showed antibiotic tolerance with 1000 times higher MBEC to tested antibiotics (Table 1).

**Table 1:** Susceptibilities to different antibiotics among planktonic and biofilms of 165 carbapenem-resistant *Klebsiella pneumoniae* clinical isolates.

Antimicrobial agents	MIC 50 (mg/L)	MIC 90 (mg/L)	MIC range (mg/L)	MBEC (mg/L)	Susceptibility		
					Resistance (n) (%)	Intermediate (n) (%)	Susceptible (n) (%)
Ceftazidime	> 512	> 512	32 to > 512	> 2048	165 (100%)	–	–
Ciprofloxacin	512	> 512	2 to > 512	> 2048	165 (100%)	–	–
Imipenem	64	256	0.25 to > 512	> 2048	156 (94.5%)	3 (1.8%)	6 (3.6%)
Meropenem	128	256	0.125 to > 512	> 2048	149 (90.3%)	4 (2.4%)	12 (7.3%)
Fosfomycin	64	> 512	2 to > 512	> 2048	52 (31.5%)	18 (10.9%)	95 (57.6%)
Amikacin	16	> 512	1 to > 512	> 2048	39 (23.6%)	32 (19.4%)	94 (57.0%)
Colistin	0.5	64	0.125 to > 512	> 2048	47 (28.50%)	–	118 (71.50%)

**Table 2:** Mechanisms of colistin resistance with respective MIC, FICI, biofilm biovolume and drug-resistant genes profiles of 47 ColRkp clinical isolates.

ColRkp isolates	Year of collection	No of isolates (n) (%)	Colistin MIC range (mg/L)	EDTA MIC range (mg/mL)	Mechanisms of colistin resistance	ESBL profile	Carbapenemase profile	Biofilm		Synergy testing		
								Biovolume	Interpretation	Colistin (mg/L) + EDTA (mg/mL)	FICI^†^	Interpretation
XDR	2016	6 (12.8%)	8–64	3–24	Disrupted *mgrB* due to insertion of IS *1 like* between Nucleotide + 55 and + 56	TEM, CTXM	NDM, OXA48	0.6072052	Strong biofilm producer	0.25 + 12	0.28125	Synergy
	2016					TEM, CTXM	NDM, OXA48	0.7851432	Strong biofilm producer		0.15625	Synergy
	2016					TEM, CTXM	OXA48	0.7547039	Strong biofilm producer		0.28125	Synergy
	2020					TEM, CTXM	OXA48	0.5837053	Strong biofilm producer		0.28125	Synergy
	2020					SHV, TEM, CTXM	NDM, VIM	0.6986195	Strong biofilm producer		0.3125	Synergy
	2021					SHV, TEM, CTXM	OXA48	0.2028673	Weak biofilm producer		0.1875	Synergy
	2016	5 (10.6%)	16–64	6–24	Disrupted *mgrB* due to insertion of IS *1 like* between Nucleotide + 71 and + 72	TEM, CTXM	NDM, OXA48	1.1295769	Strong biofilm producer		0.15625	Synergy
	2016					TEM, CTXM	OXA48	0.9330016	Strong biofilm producer		0.375	Synergy
XDR	2021	5 (10.6%)	16–64	6–24	Disrupted *mgrB* due to insertion of IS *1 like* between Nucleotide + 71 and + 72	TEM, CTXM	OXA48	0.2589766	Moderate biofilm producer	0.25 + 12	0.28125	Synergy
	2021					SHV, TEM, OXA, CTXM	NDM	0.7059172	Strong biofilm producer		0.26562	Synergy
	2021					SHV, TEM, CTXM	NDM	0.4435550	Moderate biofilm producer		0.125	Synergy
	2019	1 (2.1%)	> 512	24	Disrupted *mgrB* due to insertion of IS *1 like* between Nucleotide + 104 and + 105	TEM, CTXM	NDM, OXA48	0.1545586	Weak biofilm producer		-	-
	2019	1 (2.1%)	32	12	Disrupted *mgrB* due to insertion of IS *1 like* between Nucleotide + 105 and + 106	OXA, CTXM	NDM	3.0136653	Strong biofilm producer		0.125	Synergy
	2018	2 (4.2%)	16–64	6–24	Disrupted *mgrB* due to insertion of IS *Kpn14 like* (IS *1*) between Nucleotide + 115 and + 116	TEM, CTXM	NDM	1.5821402	Strong biofilm producer		0.51562	Synergy
	2021					TEM, CTXM	NDM	0.75079400	Strong biofilm producer		0.1875	Synergy
XDR	2018	2 (4.2%)	8–32	6–12	Disrupted *mgrB* due to insertion of IS *Kpn14 like* (IS *1*) between Nucleotide + 117 and + 118	SHV	NDM	1.7099703	Strong biofilm producer	0.25 + 12	0.5125	Synergy
	2021					SHV	NDM	0.4536482	Strong biofilm producer		0.125	Synergy
	2019	5 (10.6%)	32–64	3–24	Disruption of promoter region by IS *1 like* between Nucleotide-7 and -8 (in promoter region, upstream of *mgrB* start codon)	TEM, CTXM	NDM, OXA48	0.9063939	Strong biofilm producer		0.28125	Synergy
	2020					SHV, TEM, CTXM	NDM, OXA48, VIM	1.0445467	Strong biofilm producer		0.3125	Synergy
	2020					SHV,TEM, CTXM	NDM, OXA48, VIM	0.3334343	Moderate biofilm producer		0.1875	Synergy
	2019					TEM, CTXM	NDM, OXA48	0.5569316	Strong biofilm producer		0.26562	Synergy
	2021					TEM, CTXM	NDM, OXA48	0.6579625	Strong biofilm producer		0.28125	Synergy
XDR	2016	2 (4.2%)	16	3–24	Disrupted *mgrB* due to insertion of IS *3 like* between Nucleotide + 121 and + 122	SHV, TEM, CTXM	OXA48	0.7402832	Strong biofilm producer	0.25 + 12	0.28125	Synergy
	2021					TEM, CTXM	OXA48	1.1349370	Strong biofilm producer		0.26562	Synergy
	2021	2 (4.2%)	32–64	6–24	Disrupted *mgrB* due to insertion of IS *903* (IS *5 like*) between Nucleotide + 74 and + 75	SHV, TEM, CTXM	OXA48	0.8357722	Strong biofilm producer		0.375	Synergy
	2021					SHV, TEM, CTXM	OXA48	0.4406312	Strong biofilm producer		0.26562	Synergy
	2020	2 (4.2%)	16–32	6–12	Disrupted *mgrB* due to insertion of IS *Ecp 1*(IS *1380 like*) between Nucleotide + 124 and + 125	SHV, OXA, TEM	NDM,OXA48	0.1867919	Weak biofilm producer		0.1875	Synergy
	2021					SHV, OXA, TEM	NDM,OXA48	0.5518096	Strong biofilm producer		0.28125	Synergy
	2017	6 (12.8%)	32–64	12–24	Non-functional MgrB due to nonsense point mutation (A7T,AAA > TAA) causing premature internal stop codon in *mgrB*	TEM, CTXM	OXA48	0.9271056	Strong biofilm producer		0.1875	Synergy
	2020					TEM, CTXM	OXA48	0.6856009	Strong biofilm producer		0.375	Synergy
XDR	2021	6 (12.8%)	32–64	12–24	Non-functional MgrB due to nonsense point mutation (G59A, TGG > TGA) causing premature internal stop codon in *mgrB*	SHV, TEM, CTXM	NDM, OXA48, VIM	0.4942083	Strong biofilm producer	0.25 + 12	0.3125	Synergy
	2021					SHV, TEM, CTXM	NDM, OXA48, VIM	0.9054448	Strong biofilm producer		0.15625	Synergy
	2019				Non-functional MgrB due to nonsense point mutation in initial codon of *mgrB* (G3A,GTG > GTA)	TEM, CTXM	NDM, OXA48	0.0622649	No biofilm producer		0.125	Synergy
	2021					TEM, CTXM	NDM, OXA48	1.3065106	Strong biofilm producer		0.28125	Synergy
	2019	4 (8.5%)	16–64	3–24	Loss of *mgrB*	TEM, CTXM	NDM, OXA48	1.3887639	Strong biofilm producer		0.28125	Synergy
	2020					SHV, TEM, CTXM	NDM, VIM	0.8322573	Strong biofilm producer		0.28125	Synergy
	2020					SHV, TEM, CTXM	OXA48	0.3045529	Moderate biofilm producer		0.28125	Synergy
XDR	2020	4 (8.5%)	16–64	3–24	Loss of *mgrB*	SHV, TEM, CTXM	NDM, OXA48, VIM	0.3832816	Moderate biofilm producer	0.25 + 12	0.26562	Synergy
	2017	2 (4.25%)	16–64	12–24	Deleterious PmrB (T157P) due to nonsense point mutation (A469C, ACC > CCC)	OXA, CTXM	OXA48	1.0952269	Strong biofilm producer		0.1875	Synergy
	2019					SHV, TEM, OXA,CTXM	NDM, OXA48	1.6059169	Strong biofilm producer		0.1875	Synergy
	2016	1 (2.1%)	8	3	*mcr* 8.2 with Wild type *mgrB, pmrAB* and *phoPQ*	SHV, TEM, CTXM	–	0.1704835	Weak biofilm producer		0.1875	Synergy
	2021	2 (4.25%)	16–32	12–24	*mcr* 8.1 with Deleterious PmrB (R256G) due to nonsense point mutation (C766G, CGC > GGC), WT *mgrB, pmrA* and *phoPQ*	SHV, TEM, CTXM	NDM, OXA48	1.0103530	Strong biofilm producer		0.3125	Synergy
XDR	2021				*mcr* 1.1 with Deleterious PmrB (R256G) due to nonsense point mutation (C766G, CGC > GGC), WT *mgrB, pmrA* and *phoPQ*	SHV, TEM, OXA, CTXM	NDM	0.5127592	Strong biofilm producer	0.25 + 12	0.3125	Synergy
PDR	2019	1 (2.1%)	64	24	Point mutation (G244A, GAA > AAA) cause E82K on the response regulator domain of PhoP which effect on function of PhoP	CTXM	OXA48	1.1478433	Strong biofilm producer	0.25 + 12	0.3125	Synergy
	2020	1 (2.1%)	16		Loss of *mgrB*	SHV, TEM, CTXM	OXA48	0.0703863	No biofilm producer		0.28125	Synergy
	2021	1 (2.1%)	> 512	24	Disrupted *mgrB* due to insertion of IS *1 like* between Nucleotide + 104 and + 105	TEM, CTXM	NDM, OXA48	0.5559858	Strong biofilm producer		–	–
	2020	1 (2.1%)	8		*mcr* 1.1 with Wild type *mgrB, pmrAB* and *phoPQ*	SHV, TEM, CTXM	NDM, OXA48, VIM	1.1197109	Strong biofilm producer		0.15625	Synergy

### Both chromosomal-mediated and plasmid-mediated mechanisms were responsible for establishing colistin resistance

**Figure 1:**
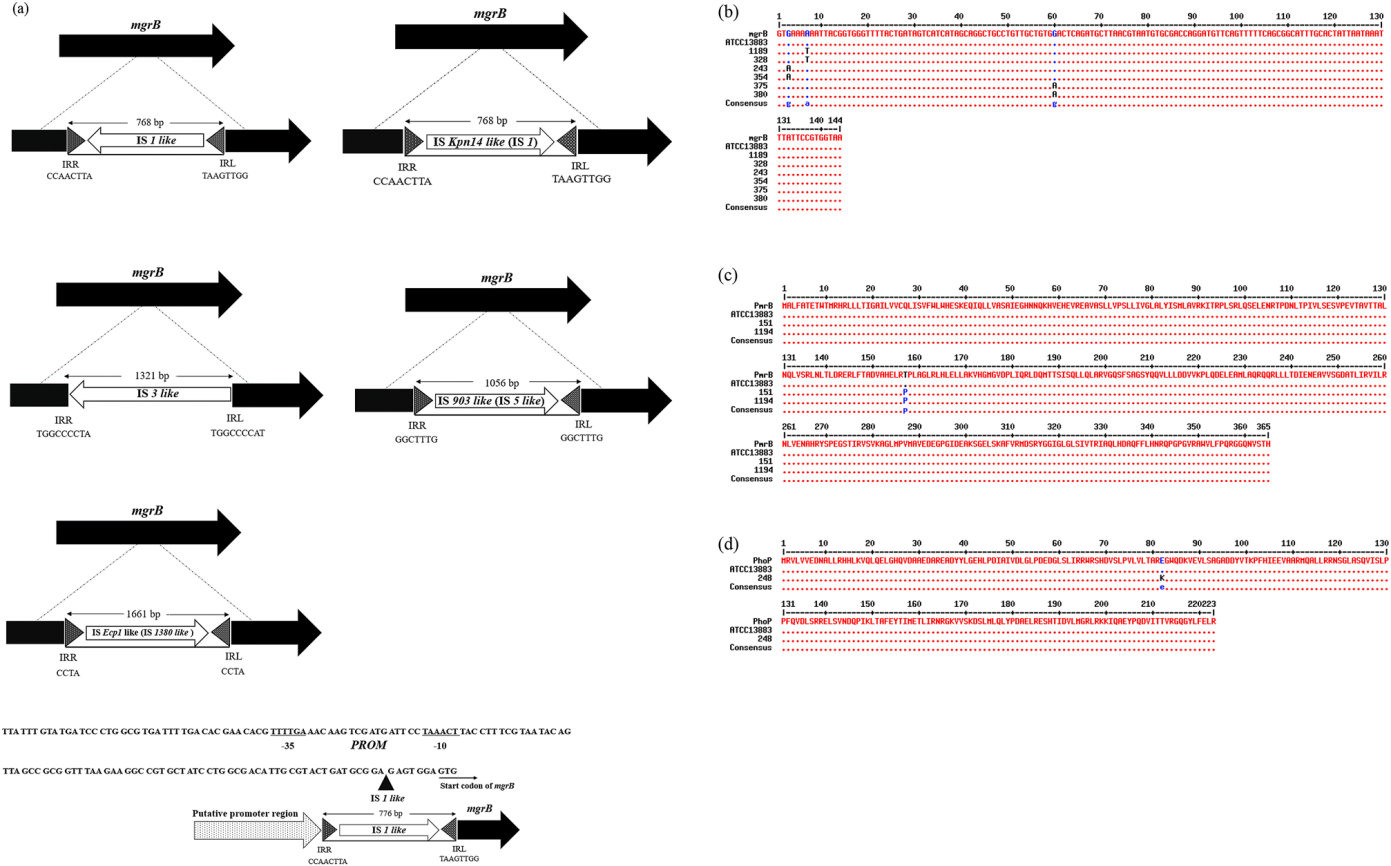
Chromosomal-mediated colistin-resistant mechanisms including (**a**) *mgrB* inactivation by different IS within the coding region and the upstream between *mgrB* start codon and putative promoter region of ColRkp (n = 29), (**b**) MgrB inactivation by point mutations—A7T MgrB, G3A MgrB, G60A MgrB in ColRkp (n = 6), (**c**) T157P PmrB in ColRkp (n = 2), (**d**) E82K PhoP in ColRkp (n = 1), observed in this study.

**Figure 2:**
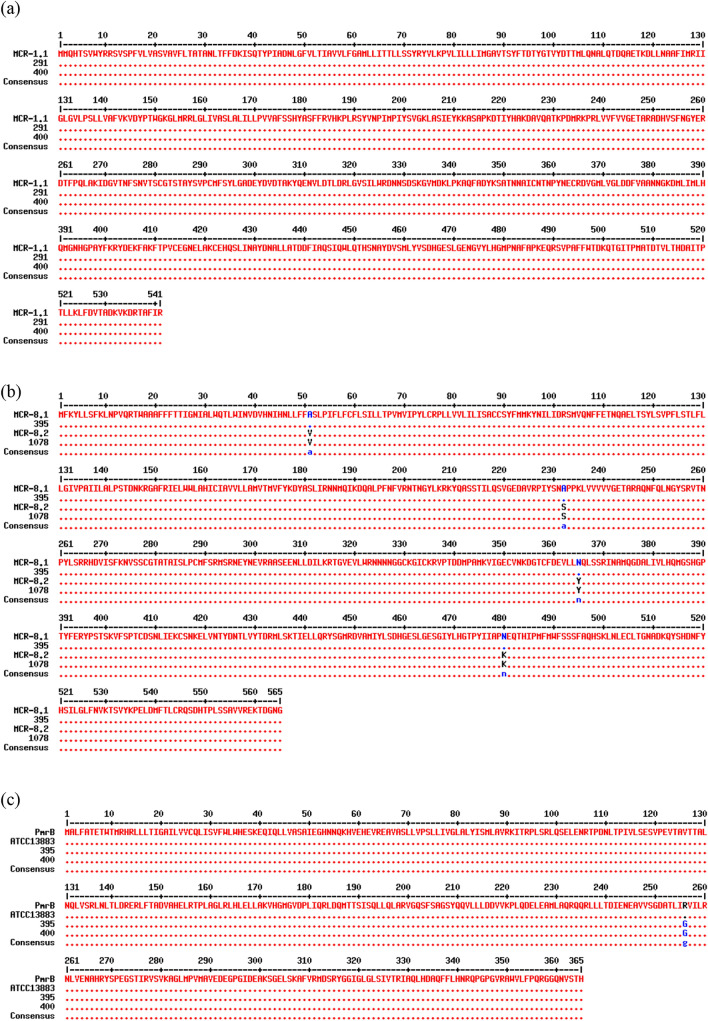
Plasmid-mediated colistin-resistant mechanisms (**a**) presence of Mcr-1.1 in ColRkp (n = 2), (**b**) presence of Mcr-8.1 and Mcr-8.2 in ColRkp (n = 1 each), (**c**) Combined presence of R256G PmrB in ColRkp (n = 2) found in this study.

Among chromosomal-mediated colistin resistance (91.5%), *mgrB* was the most inactivated chromosomal gene (85.1%) primarily by insertion sequences (IS) (61.7%) including IS*1-like*, IS*kpn14*-*like*, IS*3-like*, IS*5-like* and IS1*380*-*like* elements (IS*Ecp1-like*) (Table 2, Fig. 1a). These were found to insert with different orientations using their inverted repeats within the coding region and the upstream between *mgrB* start codon and putative promoter region (Table 2, Fig. 1a). Furthermore, different genetic alterations in the *mgrB* coding sequence (12.8%) were detected, including point mutations in the initial codon (G3A, GTG > GTA), as well as those generating an internal stop codon (A7T-AAA > TAA, G60A-TGG > TGA). We also observed deletion of *mgrB* since no *mgrB* PCR amplicons could be detected despite utilizing various primers (10.6%) in both XDR and PDR ColRkp isolates (Table 2, Fig. 1b). Point mutations in *pmrB* causing the amino acid substitutions—T157P (A469C, ACC > CCC) and *phoP*-E82K (G244A, GAA > AAA) were detected in 4.25% and 2.1% respectively (Table 2, Fig. 1c,d). Interestingly, all 47 ColRkp isolates used in this study harbored wild type *pmrA* and *phoQ* genes. The presence of plasmid-mediated *mcr* 1.1 or 8.2 alone with low level colistin resistance (8 mg/L), and the combined presence of *mcr* 1.1 or 8.1 with PmrB—R256G (C766G, CGC > GGC) demonstrating increased colistin resistance (16–32 mg/L) were investigated in 4 ColRkp isolates (8.5%) (Table 2, Fig. 2a–c). Statistically non-significant distributions of ESBLs and carbapenemases, apart from KPC and IMP, were observed in ColRkp with different colistin-resistant mechanisms (Table 2) (Supplementary results Table 2). The most common ESBLs and carbapenemase profiles were TEM-CTXM (n = 20, 46.5%) and OXA-48 (n = 15, 34.8%) in ColRkp with chromosomal-mediated colistin resistance, as well as SHV,TEM, CTX-M (n = 3, 75%) and NDM (n = 1, 25%) in ColRkp with plasmid-mediated colistin resistance, respectively (Supplementary results Table 2).

### Expressions of LPS modification genes were significantly upregulated in ColRkp isolates

**Figure 3:**
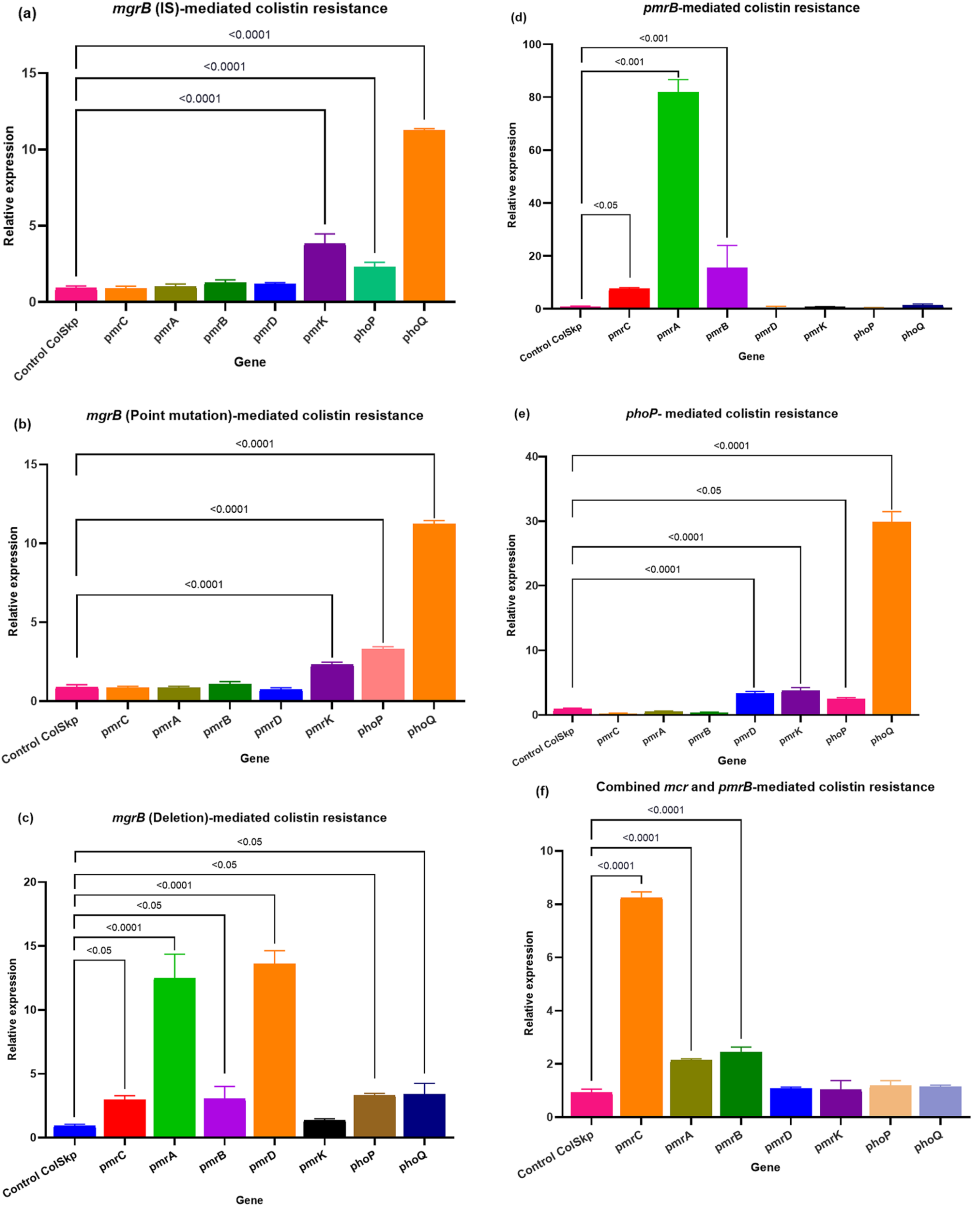
Expressions of LPS modification genes among (**a**) ColRkp with IS*1-like* integration in *mgrB*, (**b**) ColRkp with G60A *mgrB*, (**c**) ColRkp with deleted *mgrB*, (**d**) ColRkp with T157P PmrB, (**e**) ColRkp with E82K PhoP, and (**f**) ColRkp with combined presence of *mcr-8.1* and R256G PmrB.

In ColRkp isolates with inactivated *mgrB* by IS*1-like* and G60A point mutation, transcriptional levels of Ara4N-related *phoPQ* and *pmrK* were significantly increased as compared to the levels in colistin-susceptible clinical strains (ColSkp) with wild type *mgrB* (Fig. 3a,b; p < 0.0001). The ColRkp with deleted *mgrB* showed significantly upregulated Ara4N-related *phoPQ*, connector-*pmrD*, and PEtN-related *pmrCAB* transcripts (Fig. 3c; p < 0.05). In ColRkp with T157P PmrB, PEtN-related *pmrCAB* was significantly overexpressed (Fig. 3d; p < 0.001). The ColRkp with E82K PhoP had considerably upregulated *phoPQ*, *pmrD*, and *pmrK* transcriptions (Fig. 3e; p < 0.0001). In ColRkp with combined presence of *mcr-8.1* and R256G PmrB, significantly overexpressed *pmrCAB* were observed (Fig. 3f; p < 0.0001). Additionally, IS*1-like* integration in *mgrB* promoter region and G3A *mgrB* mutation significantly reduced *mgrB* expressions (p < 0.05) (Supplementary results Fig. 2). Because the majority of ColRkp had IS-mediated or point mutations in *mgrB* with significant overexpression of Ara4N-related LPS modification genes, several genetic alterations in *mgrB* with increased expressions of Ara4N-related *phoPQ* and *pmrK* were considered to be important for establishing colistin resistance in our isolates (Fig. 3a–f).

### The abilities to produce biofilms varied between XDR and PDR ColRkp isolates

**Figure 4:**
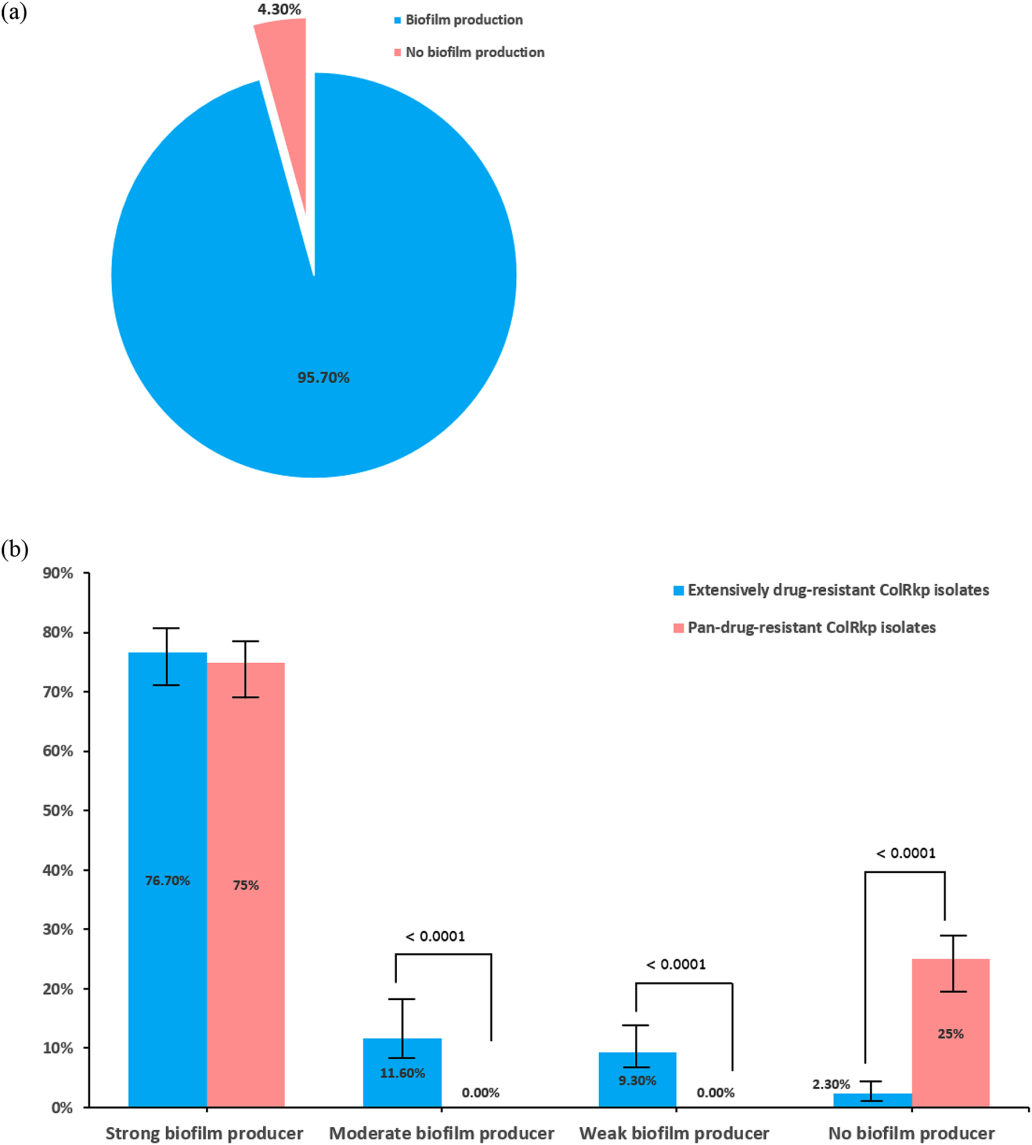
(**a**) Determination of biofilm production, (**b**) classification of biofilm producers among XDR and PDR ColRkp clinical isolates.

The majority of ColRkp isolates (95.7%) produced biofilms, with 76.7% of XDR ColRkp produced strong biofilms, 11.6% had moderate biofilms, 9.3% formed weak biofilms and 2.3% developed no biofilms (Fig. 4a,b). Meanwhile, 75% of PDR ColRkp developed strong biofilms and 25% had no biofilms (Fig. 4b). Significant associations existed between XDR and PDR ColRkp isolates in terms of moderate or weak abilities to produce biofilms, which were higher in XDR ColRkp than in PDR isolates. Interestingly, no biofilm-producing ability was higher in PDR than XDR ColRkp (Fig. 4b; p < 0.0001). It was also observed that all five colistin-susceptible *K. pneumoniae* (ColSkp) strains developed significantly stronger biofilms (100%) when compared to XDR and PDR ColRkp (Supplementary results Table 1; p < 0.0001).

### Coexistence and altered expressions of virulence factors were observed in ColRkp

**Figure 5:**
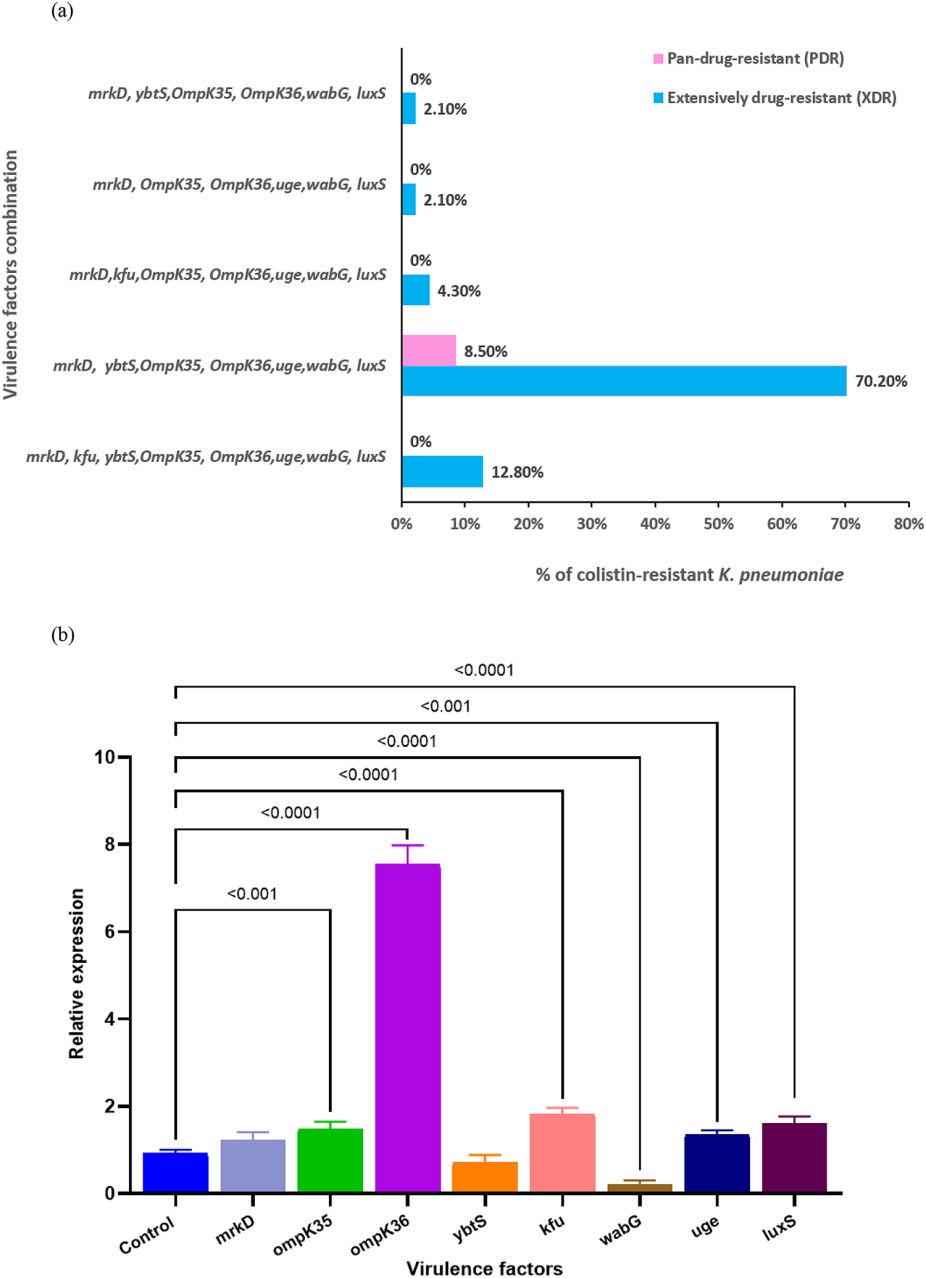
(**a**) Frequencies of virulence factors combination present among XDR and PDR ColRkp clinical isolates, (**b**) Relative expression of different virulence factors in ColRkp clinical isolates.

The existence of all tested bacterial virulence genes as *mrkD-kfu-ybtS-ompK35-ompK36-uge*-*wabG-luxS* combination was detected in 12.8% of XDR ColRkp isolates (Fig. 5a). Meanwhile, the most prevalent virulence gene combination was *mrkD-ybtS-ompK35*-*ompK36-uge-wabG*-*luxS* combination, which was observed in 70.2% of XDR strains and 8.5% of PDR strains (Fig. 5a). The combination of *mrkD-kfu-ompK35-ompK36-uge-wabG*-*luxS* was identified in 4.3% of XDR strains (Fig. 5a). The least common virulence gene combinations were *mrkD-ompK35-ompK36-uge-wabG-luxS* and *mrkD*-*ybtS-ompK35-ompK36-wabG-luxS* combinations which were observed in 2.1% each of XDR ColRkp isolates (Fig. 5a). In comparison to ColSkp clinical isolates, XDR ColRkp displayed altered expressions of virulence factors, showing significantly higher expressions of *ompK35, ompK36, kfu*, *uge*, and *luxS* (p < 0.0001), as well as significantly lower expression of *wabG* (Fig. 5b; p < 0.001).

### Colistin-EDTA combination showed remarkable synergistic effects against ColRkp in vitro

**Figure 6:**
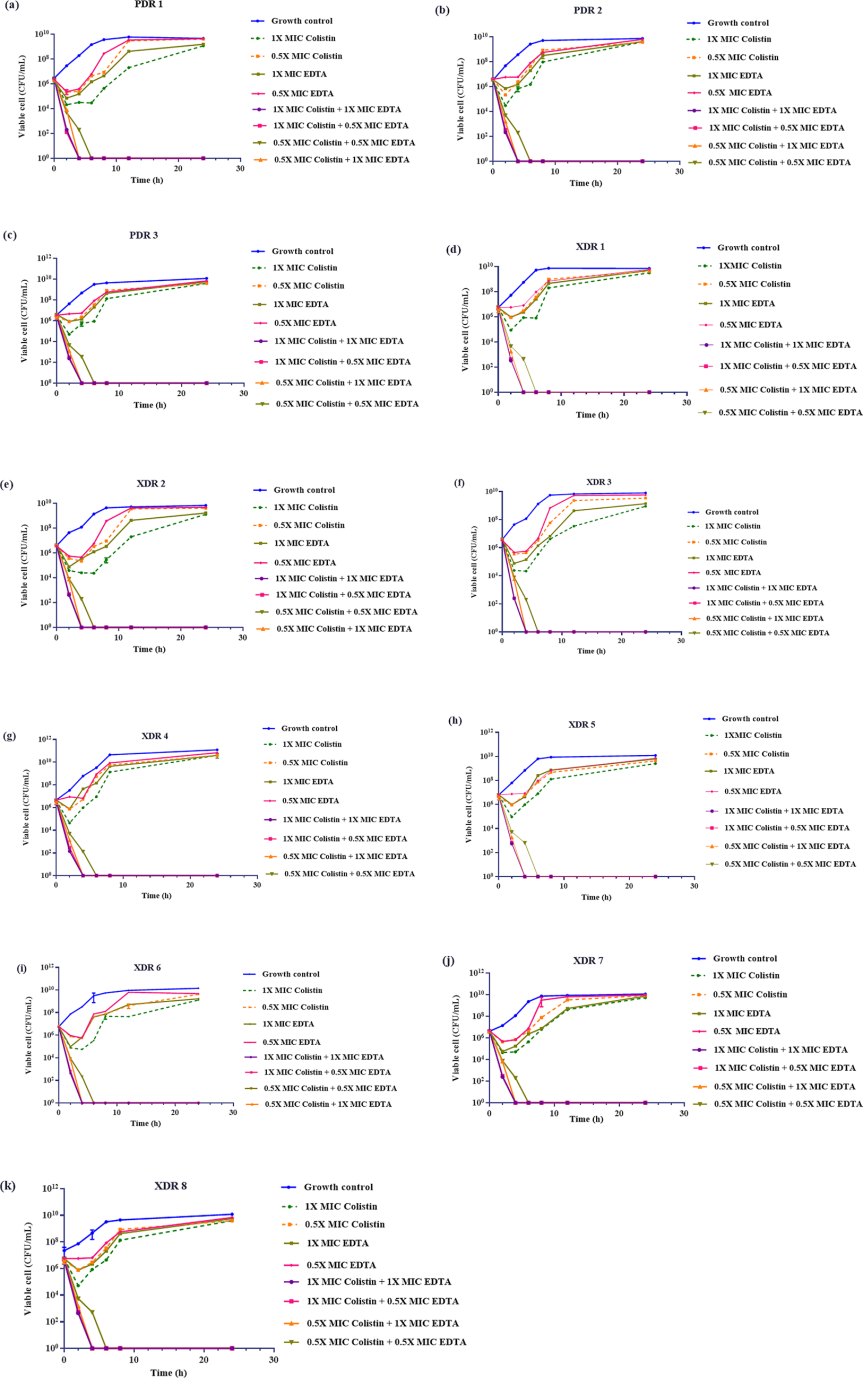
Time-kill effects of single and combination of colistin and EDTA on 11 representative PDR and XDR ColRKp clinical isolates with plasmid-mediated and chromosomal *mgrB* or *pmrB* or *phoP*-mediated colistin-resistant mechanisms.

The adjuvant-EDTA was discovered to have inhibitory effects on ColRkp at concentrations of 3-24 mg/mL (Table 2). When we performed checkerboard assays on ColRkp isolates (n = 45), colistin MIC was lowered to 0.25 mg/L when given in conjunction with 12 mg/mL EDTA, and this colistin-EDTA combination displayed substantial synergistic effects (FICI ≤ 0.5) on all tested 45 ColRkp isolates (Table 2). Using time-kill synergy confirmation assays, synergistic effects of colistin-EDTA combination were confirmed in 11 representative ColRkp with different colistin-resistant mechanisms, including 3 PDR ColRkp with *mgrB* loss, E82K PhoP, and *mcr-1.1*, and 8 XDR ColRkp with *mgrB* inactivation by IS*1-like*, IS*3-like*, IS*5-like*, IS*1380-like* elements, point mutations- G60A, A7T *mgrB*, T157P PmrB and combined *mcr-8.1* with R256G PmrB (Fig. 6a–k). Despite bacterial regrowth after 6 h of colistin and EDTA monotherapy, colistin-EDTA combination exhibited remarkable synergistic activities in reducing > 3log_10_ of bacteria starting 2 h after treatment and produced prolonged bactericidal effects with no regrowth until 24 h, in all tested XDR and PDR ColRkp isolates (n = 11), regardless of their underlying colistin resistance mechanisms (Fig. 6a–k).

### Administration of colistin-EDTA combination showed potent synergistic activities in murine ColRkp-associated bacteraemia

**Figure 7:**
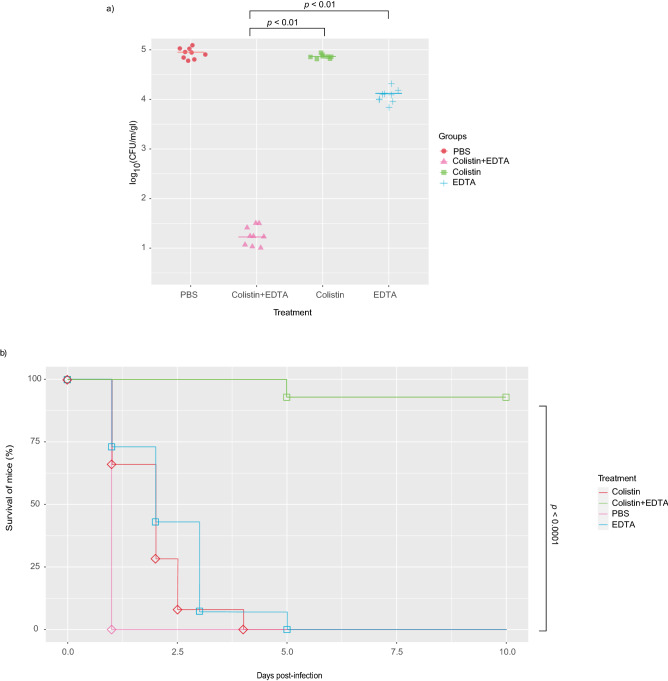
Effects of colistin, EDTA and colistin-EDTA combination therapy on (**a**) bacterial burden in murine peritoneal ColRkp-associated bacteraemia (**b**) survival of treated mice infected with ColRkp-associated bacteraemia.

When compared to colistin and EDTA monotherapy, single intraperitoneal administration of colistin-EDTA combination significantly reduced bacterial burden in murine ColRkp-associated bacteraemia induced by strong biofilm-producing XDR ColRkp with inactivated *mgrB* by IS*1-like* element with the presence of evaluated virulence factors (Fig. 7a; p < 0.01). Intraperitoneal colistin-EDTA combination therapy once a day significantly improved the survival of treated mice with ColRkp-associated bacteraemia as compared to their monotherapy (Fig. 7b; p < 0.0001).

## Discussion

*Klebsiella pneumoniae* is a pathogen causing severe untreatable hospital-acquired infections in immunocompromised patients owing to increasing rates of antimicrobial resistance^1^. With the rising prevalence of CRkp, colistin has emerged as a feasible therapeutic option due to paucity of effective therapeutic alternatives and restrictions in novel antibiotic development^3^. Consequently, the global prevalence of ColRkp has steadily increased as a result of expanded usage of colistin, revealing the significant threat for the emergence and spread of extensively and pandrug-resistant strains around the world^3^. In our study, a total of 165 CRkp isolates were observed to exhibit different antibiotic susceptibilities in both planktonic and biofilm environments. Among CRkp, we discovered a 28.5% prevalence of XDR and PDR ColRkp (n = 47), which increased over time from 14.9% in 2016 to 36.2% in 2021.The colistin-resistant rate in our clinical setting is comparable to India (30%)^25^ and Italy (22.4%)^26^, but higher than other clinical settings in Thailand (6.6%)^27^, Nigeria (9.1%)^28^ and other regions of the world^29^. Despite the lack of clinical data on colistin use in our hospital, the rising trend of ColRkp is almost certainly due to selective pressure from increased colistin use in clinical settings for increasing CRkp burden, and in poultry industry setting as a short-term colistin preventive strategy for Gram-negative bacterial infections^30,31^. This could lead to the emergence and colonization of ColRkp among patients, healthy adults, and food animals^27,30,31^, causing further circulation of colistin resistance with a higher regional colistin-resistant prevalence in Thailand.

Because colistin resistance is continuously growing and varying between countries over time^3,31^, addressing the underlying colistin-resistant mechanisms has become critical to deduce. Among ESBL and carbapenemase-producing ColRkp in this study, *mgrB* alteration played a significant role (85.1%), with inactivation by IS*1-like*, IS*kpn14-like*, IS*3-like*, IS*5-like* and IS1*380-like* elements (61.7%), point mutation (12.8%) and deletion (10.6%), which is consistent with several studies around the world^32–34^. Downregulated expressions of *mgrB* were observed in ColRkp with IS integration in promoter region and G3A *mgrB*, as proposed previously^32,35^. Moreover, as evidenced in previous studies, expressions of Ara4N-related and PEtN-related LPS modification genes were significantly upregulated in ColRkp isolates with different underlying colistin resistance mechanisms^35–37^. Because the majority of ColRkp (85.1%) had inactivated *mgrB*, several genetic alterations in *mgrB* with upregulated Ara4N-related LPS modification genes were considered to be crucial in establishing colistin resistance in our isolates, as previously demonstrated^32,35,36^. In this study, 4.25% of isolates exhibited the T157P PmrB, along with overexpression of PEtN-related *pmrCAB* operons, which has previously been proved to cause colistin resistance in *K. pneumoniae*^38^. Amino acid substitution—R256G PmrB with significant *pmrCAB* transcription, was revealed as a combined colistin-resistant mechanism in 4.25% of our isolates (n = 2) harboring *mcr*-1.1 or *mcr-*8.2 genes. Although the PROVEAN bioinformatic tool anticipated a deleterious effect of R256G PmrB on its protein function, this substitution was discovered as lineage-specific mutations in both polymyxin-susceptible as well as resistant *K. pneumoniae*, and it has been confirmed by others to be unrelated to colistin resistance in *K. pneumoniae*^39–42^. Furthermore, PhoQ E82K substitution with significant expression of *phoQ* was identified in 2.1% of ColRkp isolates, which is consistent with prior research^43^.

This study showed an 8.5% prevalence of plasmid-mediated mcr genes, which is higher than reports described in earlier studies in Thailand (< 1 to 3.2%)^41–45^. Until recently, *K. pneumoniae* of livestock origins from different regions of Thailand have been documented to harbor plasmid-mediated mcr-8 phosphoethanolamine transferase^27,41^. Our study is the first to show the presence of mcr-8.1 and mcr-8.2 in ESBL and carbapenemase-producing ColRkp isolates from human clinical samples in Thailand. These findings revealed that mcr-8 and its variants have been existing for a period and are widely disseminated among *K. pneumoniae* of both human and animal origins in this region, suggesting a growing threat of antibiotic resistance in the years^46^. The judicious use of colistin, as well as continuous monitoring of mcr genes transferability and stability, will tremendously help in the prevention and control of antimicrobial resistance^47^.

Previous studies reported the establishment of hypervirulent ColRkp with diverse virulence characteristics^8–11^ and the potential roles of *mgrB, pmrAB* and *phoPQ* in supporting bacterial virulence^19–21^, highlighting the importance of exploring the association between colistin resistance and other virulence factors that influence bacterial pathogenicity. Biofilm formation and diverse virulent factors including *mrkD*, *kfu, ybtS*, *ompK35, ompK36*, *uge*, *wabG* and *luxS* have been implicated in bacterial colonization, invasion, and pathogenicity within the host^12–18^. In this study, we discovered that majority of ColRkp produced biofilms, whereas significant associations existed between XDR and PDR ColRkp in terms of moderate, or weak biofilm-producing abilities, which were higher in XDR than in PDR isolates. Interestingly, no biofilm-producing ability was higher in PDR than XDR ColRkp. These findings suggest that not only PDR ColRkp, but XDR ColRkp may also have increased biofilm-mediated antibiotic tolerance which could enhance their abilities to resist the antibiotics effects in order to produce untreatable infections^48^. Additionally, all of the evaluated virulence genes were encoded in 12.8% of XDR ColRkp stains, and other virulence gene combinations were encoded in varying frequencies in other XDR and PDR ColRkp isolates. There were significant overexpression of *ompK35, ompK36, kfu, uge*, and *luxS* as well as lower expression of *wabG* in ColRkp compared to ColSkp, demonstrating the coexistence and altered expression of bacterial virulence factors in ColRkp. Due to their resistance to last resort colistin therapy, increased biofilm-producing abilities, coexistence and altered expression of virulence factors, these isolates would be very challenging to treat and it emphasizes the critical requirement for innovative therapy to combat these infections in healthcare settings^8,11,22^.

Consistent with earlier findings, a colistin-EDTA combination demonstrated in vitro potent synergistic effects at significantly lower colistin concentrations with no bacterial regrowth, implying lower probabilities of developing colistin toxicities under this combination therapy, and inferring fewer resistance concerns after its prolonged therapy^6^. Despite previous studies highlighting colistin-EDTA combination as lock therapy in localized catheter-associated infections^6,49^, administration of this combination in our murine bacteremia model with ColRkp resulted in a reduction of bacterial burden and increased animal survival, indicating their potent in vivo efficacy as systemic therapy in overcoming ColRkp-associated bacteremia. The potent synergistic effects of colistin-EDTA against XDR and PDR ColRkp isolates could be attributed to EDTA ions sequestration activities, which increase bacterial outer membrane permeabilities and then sensitize as well as synergize with colistin to regain colistin efficacy of increased permeabilizations, leading to enhanced intracellular content release and bacterial death^3,50^. EDTA chelation could augment colistin's entry into bacteria to exert bactericidal effects by blocking intracellular targets of colistin—essential respiratory enzymes, thereby unlocking colistin resistance regardless of underlying colistin-resistant mechanisms^51^.

In conclusion, these data revealed a rising trend of both chromosomal and plasmid-mediated colistin resistance in *K. pneumoniae* isolated from Chulalongkorn Memorial Hospital, Thailand between 2016 to 2021, which was also linked to altered bacterial virulence factors. Potent synergistic effects of colistin-EDTA combination against ColRkp-associated bacteremia suggest their promising application as systemic therapy in unlocking colistin resistance of untreatable superbugs, regardless of underlying colistin-resistant mechanisms, but more clinical trials are necessary to further evaluate their clinical efficacy, tolerance, and safety. The effects of altered virulence gene expression in ColRkp will need to be investigated further to learn more about how colistin-resistant bacteria modulate their pathogenicity inside the host, which will support the implementation of more effective targeted strategies to overcome and mitigate their infectivity.

## Materials and methods

### Bacterial isolates and antimicrobial susceptibility testing

A total of 165 CRkp clinical isolates which showed resistance to either imipenem or meropenem or both, were obtained from Chulalongkorn Memorial Hospital, Thailand during 2016 to 2021 after approved by the Institutional Review Board (IRB) of the Faculty of Medicine, Chulalongkorn University, Bangkok, Thailand. All isolates are identified by 16srRNA sequencing. To determine planktonic susceptibilities (MIC) to colistin and EDTA, drugs were serially diluted two-fold in 96-well microtiter plates using standard broth microdilution according to criteria in EUCAST (criteria for *Enterobacteriaceae* for colistin only)^52^ and CLSI^53^. Planktonic susceptibilities to other antibiotics including imipenem, meropenem, ceftazidime, ciprofloxacin, amikacin and fosfomycin supplemented with glucose-6-phosphate were determined by agar dilution^53^. MIC was determined as the lowest concentration that inhibited the visible growth of the bacteria. According to antibiotic susceptibilities, ColRkp were categorized to XDR (non-susceptibility to at least one agent in all but two or fewer antimicrobial categories) and PDR (non-susceptibility to all agents in all antimicrobial categories)^54^.

### Characterization of colistin resistance mechanisms

Chromosomal-mediated colistin resistance mechanisms were analyzed by targeted amplification and sequencing of *mgrB*, *pmrA*, *pmrB*, *phoP* and *phoQ* using previously described primers^35,38^ (Supplementary Methods). The resulting nucleotide and amino acid sequences were analyzed by Basic Local Alignment Search Tool (BLAST) and multiple sequence alignment by Florence Corpet (http://multalin.toulhouse.inra.fr/mutalin/multalin.html) was used to compare mutations observed in ColRkp nucleotide and amino acid sequences to reference sequences of *K. pneumoniae* subsp. *pneumoniae* MGH 78578 (GenBank accession number. CP_000647.1), Insertion Sequences (ISs) were analyzed using the IS finder web site (www-is.biotoul.fr). For determination of plasmid-mediated colistin resistance, *mcr*-1-9 genes were screened by PCR using primers as established previously and confirmed by subsequent sequence analysis using the primers that target for amplification of *mcr* gene of interest^55,56^. The PROVEAN tool v.1.1.5 (http://provean.jcvi.org/index.php) was used to predict the effect of amino acid substitutions on protein function^57^. PROVEAN score ≤ − 2.5 was deleterious for protein function, and a score > − 2.5 was considered to have a neutral effect on protein function.

### Determination of extended spectrum β-lactamase (ESBL) and carbapenemase genes

Presence of ESBL (CTXM,TEM,OXA,SHV) and carbapenemase genes (KPC, NDM, OXA-48, IMP, VIM) conferring resistance to broad range of β-lactam and carbapenem antibiotics were analyzed among ColRkp isolates using primers as previously reported^58,59^.

### Determination of the expressions of LPS modification genes associated with ColRkp

By using specific primers as previously described^60,61^, Quantitative RT-PCR (qRT-PCR) was used to assess the expression levels of LPS modification genes among ColRkp isolates with various colistin-resistant mechanisms. These genes include Ara4N-related *pmrK* and *phoPQ*, connector *pmrD*, and PEtN-related *pmrCAB* that are known to be involved in establishing Ara4N-related and PEtN-related LPS modification for colistin resistance. The tested isolates were ColRkp isolates with IS*1*-like, G60A and deleted *mgrB*, T157P PmrB, E82K PhoP and combined presence of *mcr-8.1* and R256G PmrB. Briefly, Monarch Total RNA Miniprep Kit (Biolabs, New England) was used to extract total RNA from tested bacterial cultures grown in Luria–Bertani broth (Merck, Darmstadt, Germany) during the mid-logarithmic growth phase. These DNase-treated purified RNA was subsequently reverse-transcribed into cDNA and qRT-PCR expression assays were performed using cDNA of tested ColRkp and ColSkp clinical isolates.

### Determination of in vitro biofilm-mediated colistin tolerance

Using crystal violet assay, the amounts of biofilms produced by ColRkp were determined for assessing their biofilm-producing abilities and biofilm-mediated antibiotic tolerance^62^. Briefly overnight cultures of bacteria were standardized with an OD_600_ of 0.02 at 600 nm (5 × 10^7^ CFU mL^−1^) and 100 μL aliquots are added in triplicate to flat-bottomed 96-well polystyrene microtiter plates (SPL Life Sciences). Plates were then incubated at 37 °C for 24 h. Adherent biofilms were fixed with crystal violet (0.1%) and stained biofilms were solubilized with 30% acetic acid. Absorbance (OD) at 560 nm was then determined using a microtiter-plate-reading fluorimeter (Varioskan Flash Multimode Reader; Thermo Fisher Scientific). All experiments were performed in triplicate and repeated three times. Minimal biofilm eradication concentration (MBEC) is the lowest concentration of antimicrobial agent that eradicates all mature biofilm biovolume and biofilm-embedded bacteria reducing bacterial viability by ≤ 10% as compared to growth controls^63,64^. Crystal violet assay was used to determine the drugs MBECs on mature ColRkp biofilms biovolume by measuring the percent eradication of biofilm biovolume^6,62–64^. Prestoblue assay was used to evaluate drugs MBECs by measuring the percent reduction of biofilm-embedded viable bacteria. In this assay, cell viability indicator—Prestoblue (Invitrogen) which turns fluorescent red in environment of viable cell was used. The fluorescence intensity of drug-treated biofilm was measured with excitation 535 nm and emission 590 nm under a microtiter-plate-reading fluorimeter^6,62–66^.

$$\mathrm{Percentage} \, \mathrm{ of} \, \mathrm{ eradication} \, \mathrm{ of} \, \mathrm{ biofilm} \, \mathrm{ biovolume}/\mathrm{viability }=\mathrm{ OD} \, \mathrm{ in } \, \mathrm{ control }-\mathrm{ OD} \, \mathrm{ in} \, \mathrm{ treatment }\times \frac{100}{\text{OD} \, \mathrm{ in } \, \mathrm{ control}}$$

### Determination of the coexistence and expressions of virulence factors in ColRkp

The presence of virulence factors (*kfu, luxS, mrkD, ompK35, ompK36, uge, wabG* and *ybtS*) were firstly identified to determine their coexistence in ColRkp, by utilizing PCR with bacterial DNA extracted by Purelink genomic DNA micro kit (Invitrogen, USA). Using previously reported specific primers and bacterial cDNA produced from mRNA isolated from bacterial cultures grown in Luria–Bertani broth during mid-logarithmic phase, qRT-PCR was then used to assess the relative expression of these 8 different virulence factors in ColRkp and ColSkp clinical isolates^67^.

### Quantitative RT-PCR (qRT-PCR)

Using the 2^∆∆CT^ method, the relative expressions of 6 different LPS-modification genes and 8 different virulence factors were computed after normalization with control housekeeping gene—*rpoD* followed by subsequent normalization against the value obtained for ColSkp isolate to evaluate and compare the fold change differences.

### Synergy assays for colistin-EDTA combination against ColRkp isolates in vitro

The synergistic activities of colistin-EDTA combination against ColRkp clinical isolates were first determined by checkerboard assay^68^. The synergistic activities were interpreted according to fractional inhibitory concentration index (FICI): Synergy: FIC index ≤ 0.5 and Additive: 0.5 > FIC index ≤ 1. The synergistic activities were then confirmed using time-kill assays against 11 representative PDR and XDR ColRkp isolates with various underlying colistin-resistant mechanisms^68^. Briefly, adjusted bacterial suspension of each ColRkp isolate was added to into each flask of 9 different growth conditions, including no drug (growth control), 1× and 0.5×MIC of colistin and EDTA as monotherapy and combination therapy, and incubated for 24 h at 37 °C. The viable cell counts (CFU/mL) under different growth conditions were then determined at 0, 2, 4, 6, 8, 12, and 24 h by plate counting. The synergistic activities are interpreted when there is ≥ 2 log10 (CFU/mL)-fold decrease in combination compared with the single antibiotic; Bactericidal activity is defined as a ≥ 3 log10 (CFU/mL)-fold decrease when compared to the number of viable cells at initial time point^68^.

### Animal study

6–8-week-old C57BL/6 male mice were purchased from Nomura Siam International (Pathumwan, Bangkok, Thailand) and were used in all experiments. Animals were at rest for 1 week in the animal facility before use. Animals received food and water ad libitum and were housed at a maximum of 2 mice per cage, weighed and closely monitored for any signs of distress throughout experimental periods. The animal study was conducted according to guidelines and protocols approved by the Institutional Animal Care and Use Committee of the Faculty of Medicine, Chulalongkorn University, Bangkok, Thailand, based on the National Institutes of Health (NIH), USA.

### In vivo murine bacteraemia model

To establish ColRkp-associated bacteremia in vivo, the previously published murine bacteremia model through intraperitoneal inoculation was performed^69^. We used a clinical XDR ColRkp isolate with the presence of evaluated virulence factors collected from the blood of bacteremia patients. This ESBL and carbapenemase-producing ColRkp also had inactivated *mgrB* from IS*1-like* insertion between nucleotides + 71 and + 72, which was one of the most common colistin-resistant mechanisms observed in this study. Immunocompetent male C57BL/6 mice were inoculated intraperitoneally with 1 × 10^6^ CFU of bacterial suspension with 5% porcine mucin (Sigma-Aldrich) and murine ColRkp-associated bacteremia was allowed to develop for 1 h as previously reported^69^. To evaluate in vivo effects of colistin, EDTA and colistin-EDTA combination, the animals with ColRkp-associated bacteremia were given a single dose of PBS (control), colistin (20 mg/kg), EDTA (40 mg/kg) and colistin-EDTA (20 mg/kg + 40 mg/kg) intraperitoneally, for a total of 4 groups with 10 animals in each group. All mice were then euthanized at 14 h post-infection. Peritoneal fluid was collected by injecting 2 mL sterile saline solution into the peritoneum, followed by gentle massage and aspiration. Peritoneal fluid samples were then serially diluted and plated on nutrient agar for counting of bacterial load. For analysis of survival in mice, the same treatment procedure was repeated once daily, and survival of mice were monitored until clinical endpoint or experimental endpoint was reached. Clinical endpoint was determined using a five-point body condition score analysing weight loss, decrease in body temperature, respiratory distress, hampered mobility, and hunched posture. Experimental endpoint was defined as 10 days post infection for mice not reaching clinical endpoint.

### Data analysis

All statistical analysis was conducted using R statistic package^70^. Data were compared by either unpaired two-tailed Student’s t-test or unpaired two-tailed Mann–Whitney’s U test. Statistical significance was accepted at *p* < 0.05, *p* < 0.01, *p* < 0.001, and *p* < 0.0001.

### Ethics approval

The study protocol was approved by the Institutional Review Board (IRB) of the Faculty of Medicine, Chulalongkorn University, Bangkok, Thailand (COA No. 045/2020, IRB No. 774/63) was performed in accordance with the ethical standards as laid down in the 1964 Declaration of Helsinki and its later amendments and comparable ethical standards. Animal care and use protocol are based upon the National Institutes of Health (NIH), USA. The protocol was approved by the Institutional Animal Care and Use Committee of the Faculty of Medicine, Chulalongkorn University, Bangkok, Thailand (Certificate No- 033/2563, Research Project No—020/2563). The study was carried out in compliance with the ARRIVE guidelines (Animal Research: Reporting of In Vivo Experiments).

### Informed consent

For this retrospective study of anonymous clinical isolates, the requirement for informed consent from patients was waived by Institutional Review Board (IRB) of the Faculty of Medicine, Chulalongkorn University, Bangkok, Thailand (COA No. 045/2020, IRB No. 774/63).

## Supplementary Information

Supplementary Information 1.

Supplementary Information 2.

## Data Availability

The authors confirm that the data supporting the findings of this study are available within the article and its additional information.
