# Supplementary material for: High prevalence of mgrB-mediated colistin resistance among carbapenem-resistant Klebsiella pneumoniae is associated with biofilm formation, and can be overcome by colistin-EDTA combination therapy
Source: Sci Rep. 2022 Jul 28;12:12939. doi: 10.1038/s41598-022-17083-5 (PMC9334626; doi:10.1038/s41598-022-17083-5)
Supplement: Supplementary file 1 — Supplementary Information 1. [file 41598_2022_17083_MOESM1_ESM.docx]

**Supplementary Results**

**High prevalence of *mgrB* medicated colistin resistance with biofilm formation among carbapenem-resistant *Klebsiella pneumoniae* overcome by colistin-EDTA combination therapy**

Aye Mya Sithu Shein^1,2,3,†^, Dhammika Leshan Wannigama^1,2,4,5,6 *^, Paul G. Higgins^8,9, †^,Cameron Hurst^6,10, †^, Shuichi Abe^4,6 †^, Parichart Hongsing^6,11,12, †^, Naphat Chantaravisoot^13,14^,Thammakorn Saethang^15^, Sirirat Luk-in^16^, Tingting Liao ^6,17,18^, Sumanee Nilgate^1,2^, Ubolrat Rirerm^1,2^, Naris Kueakulpattana ^1,2^, , Sukrit Srisakul^1,2^, Apichaya Aryukarn^1,2^, Matchima Laowansiri^1,2^, Lee Yin Hao^1,2^, Manta Yonpiam^1,2^, Naveen Kumar Devanga Ragupathi^4, 6, 19,20^, Teerasit Techawiwattanaboon ^1, 21^, Natharin Ngamwongsatit ^22^, Mohan Amarasiri^6,23^, Puey Ounjai ^24^**,** Rosalyn Kupwiwat^6,25^, Phatthranit Phattharapornjaroen^6,26,27^, Vishnu Nayak Badavath ^28^, Asada Leelahavanichkul^1,29^, Anthony Kicic ^30.31.32,33^, Tanittha Chatsuwan^1,2,*^

^1^ Department of Microbiology, Faculty of Medicine, Chulalongkorn University, King Chulalongkorn Memorial Hospital, Thai Red Cross Society, Bangkok, Thailand.

^2^ Center of Excellence in Antimicrobial Resistance and Stewardship, Faculty of Medicine, Chulalongkorn University, Bangkok, Thailand.

^3^​Interdisciplinary Program of Medical Microbiology, Graduate School, Chulalongkorn University, Bangkok, Thailand.

^4^Department of Infectious Diseases and Infection Control, Yamagata Prefectural Central Hospital, Yamagata, Japan.

^5^ Biofilms and Antimicrobial Resistance Consortium of ODA receiving countries, The University of Sheffield, Sheffield, United Kingdom

^6^ School of Medicine, Faculty of Health and Medical Sciences, The University of Western Australia, Nedlands, Western Australia, Australia.

^7^ Pathogen Hunter's Research Collaborative Team, Department of Infectious Diseases and Infection Control, Yamagata Prefectural Central Hospital, Yamagata, Japan.

^8^ Institute for Medical Microbiology, Immunology and Hygiene, Faculty of Medicine and University Hospital Cologne, University of Cologne, Cologne, Germany.

^9^ German Centre for Infection Research, Partner site Bonn-Cologne, Cologne, Germany.

^10^ Molly Wardaguga Research Centre, Charles Darwin University, Queensland, Australia.

^11^ Mae Fah Luang University Hospital, Chiang Rai, Thailand.

^12^ School of Integrative Medicine, Mae Fah Luang University, Chiang Rai, Thailand.

^13^ Department of Biochemistry, Faculty of Medicine, Chulalongkorn University, Bangkok, Thailand.

^14^ Center of Excellence in Systems Biology, Research Affairs, Faculty of Medicine, Chulalongkorn University, Bangkok, Thailand.

^15^ Department of Computer Science, Faculty of Science, Kasetsart University, Bangkok, Thailand.

^16^ Department of Clinical Microbiology and Applied Technology, Faculty of Medical Technology, Mahidol University, Bangkok, Thailand.

^17^ Department of Physiology, Faculty of Medicine, Chulalongkorn University, Bangkok, Thailand.

^18^ Center of Excellence for Microcirculation, Faculty of Medicine, Chulalongkorn University

^19^ Department of Chemical and Biological Engineering, The University of Sheffield, Sheffield, United Kingdom

^20^ Department of Clinical Microbiology, Christian Medical College, Vellore, India

^21^ Chula Vaccine Research Center, Faculty of Medicine, Chulalongkorn University, Bangkok, Thailand

^22^ Department of Clinical Sciences and Public Health, Faculty of Veterinary Science, Mahidol University, Nakhon Pathom, Thailand

^23^ Laboratory of Environmental Hygiene, Department of Health Science, School of Allied Health Sciences, Kitasato University, Kitasato, Sagamihara-Minami, Kanagawa, 252-0373, Japan.

^24^ Department of Biology, Faculty of Science, Mahidol University, Bangkok, Thailand

^25^ Department of Dermatology. Faculty of Medicine Siriraj Hospital. Mahidol University, Bangkok, Thailand.

^26^ Department of Emergency Medicine, Center of Excellence, Faculty of Medicine Ramathibodi Hospital, Mahidol University, Bangkok, Thailand

^27^ Institute of Clinical Sciences, Department of Surgery, Sahlgrenska Academy, Gothenburg University, 40530 Gothenburg, Sweden

^28^ School of Pharmacy & Technology Management, SVKM's Narsee Monjee Institute of Management Studies (NMIMS), Hyderabad, 509301, India

^29^ Translational Research in Inflammation and Immunology Research Unit (TRIRU), Department of Microbiology, Chulalongkorn University, Bangkok, Thailand

^30^ Telethon Kids Institute, University of Western Australia, Nedlands, 6009, Western Australia, Australia.

^31^ Centre for Cell Therapy and Regenerative Medicine, Medical School, The University of Western Australia, Nedlands, 6009, Western Australia, Australia.

^32^ Department of Respiratory and Sleep Medicine, Perth Children’s Hospital, Nedlands, 6009, Western Australia, Australia.

^33^ School of Public Health, Curtin University, Bentley, 6102, Western Australia, Australia.

^†^These authors contributed equally to this work

*Co-Corresponding Author: Dhammika Leshan Wannigama and Tanittha Chatsuwan,

**Supplementary Figure 1.**

Rising trends of ColRkp clinical isolates collected between 2016-2021.

**Supplementary Table 1.**

Biofilm biovolume of colistin-susceptible *K. pneumoniae* clinical isolates in this study.

**Supplementary Figure 1**


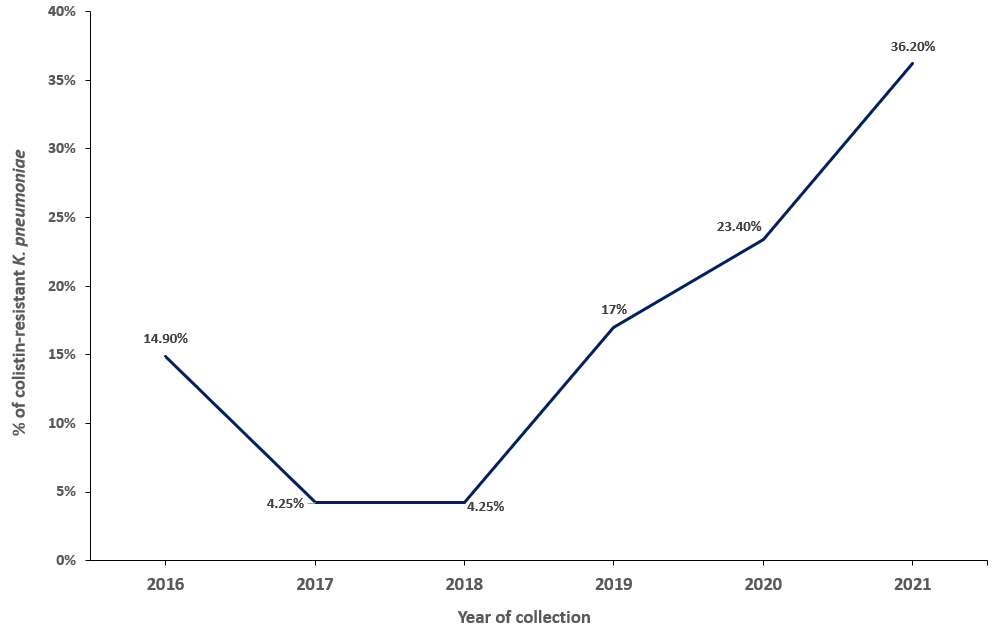


**Supplementary Table 1**

| **Colistin-susceptible Strains** | **Biofilm biovolume (OD560nm)** | **Interpretation** | **P value** |
| --- | --- | --- | --- |
| ColSkp 1 | 1.249813 | Strong biofilm producer | <0.0001 |
| ColSkp 2 | 1.428657 | Strong biofilm producer |  |
| ColSkp 3 | 0.62452 | Strong biofilm producer |  |
| ColSkp 4 | 0.975548 | Strong biofilm producer |  |
| ColSkp 5 | 0.588569 | Strong biofilm producer |  |
