# Supplementary material for: High prevalence of mgrB-mediated colistin resistance among carbapenem-resistant Klebsiella pneumoniae is associated with biofilm formation, and can be overcome by colistin-EDTA combination therapy
Source: Sci Rep. 2022 Jul 28;12:12939. doi: 10.1038/s41598-022-17083-5 (PMC9334626; doi:10.1038/s41598-022-17083-5)
Supplement: Supplementary file 2 — Supplementary Information 2. [file 41598_2022_17083_MOESM2_ESM.docx]

**Supplementary Methods**

**High prevalence of *mgrB* medicated colistin resistance with biofilm formation among carbapenem-resistant *Klebsiella pneumoniae* overcome by colistin-EDTA combination therapy**

Aye Mya Sithu Shein^1,2,3,†^, Dhammika Leshan Wannigama^1,2,4,5,6 *^, Paul G. Higgins^8,9, †^,Cameron Hurst^6,10, †^, Shuichi Abe^4,6 †^, Parichart Hongsing^6,11,12, †^, Naphat Chantaravisoot^13,14^,Thammakorn Saethang^15^, Sirirat Luk-in^16^, Tingting Liao ^6,17,18^, Sumanee Nilgate^1,2^, Ubolrat Rirerm^1,2^, Naris Kueakulpattana ^1,2^, , Sukrit Srisakul^1,2^, Apichaya Aryukarn^1,2^, Matchima Laowansiri^1,2^, Lee Yin Hao^1,2^, Manta Yonpiam^1,2^, Naveen Kumar Devanga Ragupathi^4, 6, 19,20^, Teerasit Techawiwattanaboon ^1, 21^, Natharin Ngamwongsatit ^22^, Mohan Amarasiri^6,23^, Puey Ounjai ^24^**,** Rosalyn Kupwiwat^6,25^, Phatthranit Phattharapornjaroen^6,26,27^, Vishnu Nayak Badavath ^28^, Asada Leelahavanichkul^1,29^, Anthony Kicic ^30.31.32,33^, Tanittha Chatsuwan^1,2,*^

^1^ Department of Microbiology, Faculty of Medicine, Chulalongkorn University, King Chulalongkorn Memorial Hospital, Thai Red Cross Society, Bangkok, Thailand.

^2^ Center of Excellence in Antimicrobial Resistance and Stewardship, Faculty of Medicine, Chulalongkorn University, Bangkok, Thailand.

^3^​Interdisciplinary Program of Medical Microbiology, Graduate School, Chulalongkorn University, Bangkok, Thailand.

^4^Department of Infectious Diseases and Infection Control, Yamagata Prefectural Central Hospital, Yamagata, Japan.

^5^ Biofilms and Antimicrobial Resistance Consortium of ODA receiving countries, The University of Sheffield, Sheffield, United Kingdom

^6^ School of Medicine, Faculty of Health and Medical Sciences, The University of Western Australia, Nedlands, Western Australia, Australia.

^7^ Pathogen Hunter's Research Collaborative Team, Department of Infectious Diseases and Infection Control, Yamagata Prefectural Central Hospital, Yamagata, Japan.

^8^ Institute for Medical Microbiology, Immunology and Hygiene, Faculty of Medicine and University Hospital Cologne, University of Cologne, Cologne, Germany.

^9^ German Centre for Infection Research, Partner site Bonn-Cologne, Cologne, Germany.

^10^ Molly Wardaguga Research Centre, Charles Darwin University, Queensland, Australia.

^11^ Mae Fah Luang University Hospital, Chiang Rai, Thailand.

^12^ School of Integrative Medicine, Mae Fah Luang University, Chiang Rai, Thailand.

^13^ Department of Biochemistry, Faculty of Medicine, Chulalongkorn University, Bangkok, Thailand.

^14^ Center of Excellence in Systems Biology, Research Affairs, Faculty of Medicine, Chulalongkorn University, Bangkok, Thailand.

^15^ Department of Computer Science, Faculty of Science, Kasetsart University, Bangkok, Thailand.

^16^ Department of Clinical Microbiology and Applied Technology, Faculty of Medical Technology, Mahidol University, Bangkok, Thailand.

^17^ Department of Physiology, Faculty of Medicine, Chulalongkorn University, Bangkok, Thailand.

^18^ Center of Excellence for Microcirculation, Faculty of Medicine, Chulalongkorn University

^19^ Department of Chemical and Biological Engineering, The University of Sheffield, Sheffield, United Kingdom

^20^ Department of Clinical Microbiology, Christian Medical College, Vellore, India

^21^ Chula Vaccine Research Center, Faculty of Medicine, Chulalongkorn University, Bangkok, Thailand

^22^ Department of Clinical Sciences and Public Health, Faculty of Veterinary Science, Mahidol University, Nakhon Pathom, Thailand

^23^ Laboratory of Environmental Hygiene, Department of Health Science, School of Allied Health Sciences, Kitasato University, Kitasato, Sagamihara-Minami, Kanagawa, 252-0373, Japan.

^24^ Department of Biology, Faculty of Science, Mahidol University, Bangkok, Thailand

^25^ Department of Dermatology. Faculty of Medicine Siriraj Hospital. Mahidol University, Bangkok, Thailand.

^26^ Department of Emergency Medicine, Center of Excellence, Faculty of Medicine Ramathibodi Hospital, Mahidol University, Bangkok, Thailand

^27^ Institute of Clinical Sciences, Department of Surgery, Sahlgrenska Academy, Gothenburg University, 40530 Gothenburg, Sweden

^28^ School of Pharmacy & Technology Management, SVKM's Narsee Monjee Institute of Management Studies (NMIMS), Hyderabad, 509301, India

^29^ Translational Research in Inflammation and Immunology Research Unit (TRIRU), Department of Microbiology, Chulalongkorn University, Bangkok, Thailand

^30^ Telethon Kids Institute, University of Western Australia, Nedlands, 6009, Western Australia, Australia.

^31^ Centre for Cell Therapy and Regenerative Medicine, Medical School, The University of Western Australia, Nedlands, 6009, Western Australia, Australia.

^32^ Department of Respiratory and Sleep Medicine, Perth Children’s Hospital, Nedlands, 6009, Western Australia, Australia.

^33^ School of Public Health, Curtin University, Bentley, 6102, Western Australia, Australia.

^†^These authors contributed equally to this work

*Co-Corresponding Author: Dhammika Leshan Wannigama and Tanittha Chatsuwan,

**Supplementary Table S 1.**

Primers for amplification of *mgrB*, *pmrAB* and *phoPQ* genes involved in chromosomal-mediated colistin resistance used in this study.

| **Target gene** | **Primer**  **name** | **DNA sequence (5’ to 3’)** | **References** |
| --- | --- | --- | --- |
| *mgrB* | mgrB_ext_F | AAGGCGTTCATTCTACCACC | 1,2 |
|  | mgrB_ext_R | TTAAGAAGGCCGTGCTATCC |  |
|  | EE_mgrB_F | GGCTATGGCGAGGATAATGAG |  |
|  | EE_mgrB_R | GCTGTGATGTAAGCGTCTGGTG |  |
|  | Int_mgrB_F | CGGTGGGTTTTACTGATAGTCA |  |
|  | Int_mgrB_R | ATAGTGCAAATGCCGCTGA |  |
| *pmrA* | pmrA ext F | CAT TTC CGC GCA CTG TCT GC |  |
|  | pmrA ext R | CAG GTT TCA GTT GCA AAC AG |  |
| *pmrB* | pmrB-F1 | GCGAAAAGATTGGCAAATCG |  |
|  | pmrB-R1 | GGAAATGCTGGTGGTCATCTGA |  |
|  | pmrB-F2 | CCCTGAATCAGTTGGTTTC |  |
|  | pmrB-R2 | ATCAATGGGTGCTGACGTT |  |
| *phoP* | phoP ext F | GAG CTT CAG ACT ACT ATC GA |  |
|  | phoP ext R | GGG AAG ATA TGC CGC AAC AG |  |
| *phoQ* | phoQ ext F | ATA CCC ACA GGA CGT CAT CA |  |
|  | phoQ ext R | CAG GTG TCT GAC AGG GAT TA |  |

**Supplementary Table S 2.**

Primers for amplification of *mcr* 1-9 genes involved in plasmid-mediated colistin resistance used in this study.

| **Target gene** | **Primer**  **name** | **DNA sequence (5’ to 3’)** | **References** |
| --- | --- | --- | --- |
| *mcr-1* | mcr-1-F | AAAGACGCGGTACAAGCAAC | 3,4 |
|  | mcr-1-R | GCTGAACATGCACGGCACAG |  |
| *mcr-2* | mcr-2-F | CGACCAAGCCGAGTCTAAGG |  |
|  | mcr-2-R | CAACTGCGACCAACACACTT |  |
| *mcr-3* | mcr-3-F | ACCTCCAGCGTGAGATTGTTCCA |  |
|  | mcr-3-R | GCGGTTTCACCAACGACCAGAA |  |
| *mcr-4* | mcr-4-F | AGAATGCCACTCGTAACCCG |  |
|  | mcr-4-R | GCGAGGATCATAGTCTGCCC |  |
| *mcr-5* | mcr-5-F | CTGTGGCCAGTCATGGATGT |  |
|  | mcr-5-R | CGAATGCCCGAGATGACGTA |  |
| *mcr-6* | mcr-6-F | AGCTATGTCAATCCCGTGAT |  |
|  | mcr-6-R | ATTGGCTAGGTTGTCAATC |  |
| *mcr-7* | mcr-7-F | GCCCTTCTTTTCGTTGTT |  |
|  | mcr-7-R | GGTTGGTCTCTTTCTCGT |  |
| *mcr-8* | mcr-8-F | TCAACAATTCTACAAAGCGTG |  |
|  | mcr-8-R | AATGCTGCGCGAATGAAG |  |
| *mcr-9* | mcr-9-F | TTCCCTTTGTTCTGGTTG |  |
|  | mcr-9-R | GCAGGTAATAAGTCGGTC |  |

**Supplementary Table S 3.**

Primers for amplification of carbapenemase and ESBL genes used in this study.

| **Target gene** | **Primer**  **name** | **DNA sequence (5’ to 3’)** | **References** |
| --- | --- | --- | --- |
| *KPC* | KPC-F | CGTCTAGTTCTGCTGTCTTG | 5,6 |
|  | KPC-R | CTTGTCATCCTTGTTAGGCG |  |
| *NDM* | NDM-F | GGTTTGGCGATCTGGTTTTC |  |
|  | NDM-R | CGGAATGGCTCATCACGATC |  |
| *OXA48* | OXA48-F | GCGTGGTTAAGGATGAACAC |  |
|  | OXA48-R | CATCAAGTTCAACCCAACCG |  |
| *VIM* | VIM-F | GATGGTGTTTGGTCGCATA |  |
|  | VIM-R | CGAATGCGCAGCACCAG |  |
| *IMP* | IMP-F | GGAATAGAGTGGCTTAAYTCT |  |
|  | IMP-R | CCAAACYACTASGTTATCT |  |
| *VEB* | VEB-A | CCTTTTGCCTAAAACGTGGA |  |
|  | VEB-A | TGCATTTGTTCTTCGTTTGC |  |
| *SHV* | SHV-F | AGGATTGACTGCCTTTTTG |  |
|  | SHV-R | ATTTGCTGATTTCGCTCG |  |
| *TEM* | TEM-C | ATCAGCAATAAACCAGC |  |
|  | TEM-H | CCCCGAAGAACGTTTTC |  |
| *CTXM* | CTX-MA | CGCTTTGCGATGTGCAG |  |
|  | CTX-MB | ACCGCGATATCGTTGGT |  |
| *OXA* | OXA-F | ATATCTCTACTGTTGCATCTCC |  |
|  | OXA-R | AAACCCTTCAAACCATCC |  |

**Supplementary Table S 4.**

Primers for amplification of *pmrCAB*, *pmrK*, *pmrD*, *phoPQ* genes used in this study.

| **Target gene** | **Primer**  **name** | **DNA sequence (5’ to 3’)** | **References** |
| --- | --- | --- | --- |
| *pmrC* | pmrC int F | GCG TGA TGA ATA TCC TCA CCA | 7,8 |
|  | pmrC int R | CAC GCC AAA GTT CCA GAT GA |  |
| *pmrA* | pmrA int F | GAT GAA GAC GGG CTG CAT TT |  |
|  | pmrA int R | ACC GCT AAT GCG ATC CTC AA |  |
| *pmrB* | pmrB int F | TGC CAG CTG ATA AGC GTC TT |  |
|  | pmrB int R | TTC TGG TTG TTG TGC CCT TC |  |
| *pmrD* | pmrD int F | GAT CGC AGA GAT TGA AGC CT |  |
|  | pmrD int R | GCG TTG CGG ATC TTC AAA GT |  |
| *pmrE* | pmrE int F | GCA TAC CGT AAT GCC GAC TA |  |
|  | pmrE int R | GGG TTG ATC TCT GTG ACA TC |  |
| *pmrK* | pmrK int F | AGT ATC GGT CAG TGG CTG TT |  |
|  | pmrK int R | CCG CTT ATC ACG AAA GAT CC |  |
| *phoP* | phoP int F | GCG TCA CCA CCT CAA AGT TC |  |
|  | phoP int R | GGC GAT ATC CGG GAG ATG TT |  |
| *phoQ* | phoQ int F | CTC AAG CGC AGC TAT ATG GT |  |
|  | phoQ int R | TCT TTG GCC AGC GAC TCA AT |  |
| *rpoD* | rpoD-for | TCCGGTGCATATGATTGAGA |  |
|  | rpoD -rev | ATACGCTCAGCCAGCTCTTC |  |

**Supplementary Table S 5.**

Primers for amplification of virulence factors used in this study.

| **Target genes** | **Primer**  **name** | **Primers’ sequences (5′ to 3′)** | **References** |
| --- | --- | --- | --- |
| *16srRNA* | 16srRNA - for | AGAGTTTGATCCTGGCTCAG | 9-11 |
|  | 16srRNA - rev | GGTTACCTTGTTACGACTT |  |
| *kfu* | kfu - for | GGCCTTTGTCCAGAGCTACG |  |
|  | kfu - rev | GGGTCTGGCGCAGAGTATGC |  |
| *ybtS* | ybtS - for | GACGGAAACAGCACGGTAAA |  |
|  | ybtS- rev | GAGCATAATAAGGCGAAAGA |  |
| *mrkD* | mrkD - for | AAGCTATCGCTGTACTTCCGGCA |  |
|  | mrkD - rev | GGCGTTGGCGCTCAGATAGG |  |
| *luxS* | luxS - for | AGTGATGCCGGAACGCGG |  |
|  | luxS - rev | CGGTGTACCAATCAGGCTC |  |
| *ompK35* | ompK35 - for | GCAATATTCTGGCAGTGGTGATC |  |
|  | ompK35 - rev | ACCATTTTTCCATAGAAGTCCAGT |  |
| *ompK36* | ompK36 - for | TTAAAGTACTGTCCCTCCTGG |  |
|  | ompK36 - rev | TCAGAGAAGTAGTGCAGACCGTCA |  |
| *uge* | uge - for | TCTTCACGCCTTCCTTCACT |  |
|  | uge - rev | GATCATCCGGTCTCCCTGTA |  |
| *wabG-* | wabG- for | ACCATCGGCCATTTGATAGA |  |
|  | wabG - rev | CGGACTGGCAGATCCATATC |  |

1 Cannatelli, A. *et al.* MgrB inactivation is a common mechanism of colistin resistance in KPC-producing *Klebsiella pneumoniae* of clinical origin. *Antimicrobial agents and chemotherapy* **58**, 5696-5703 (2014).

2 Jayol, A. *et al.* Resistance to colistin associated with a single amino acid change in protein PmrB among *Klebsiella pneumoniae* isolates of worldwide origin. *Antimicrobial agents and chemotherapy* **58**, 4762-4766 (2014).

3 Borowiak, M. *et al.* Identification of a novel transposon-associated phosphoethanolamine transferase gene, *mcr*-5, conferring colistin resistance in d-tartrate fermenting *Salmonella enterica* subsp. *enterica* serovar *Paratyphi B*. *Journal of Antimicrobial Chemotherapy* **72**, 3317-3324 (2017).

4 Tolosi, R. *et al.* Rapid detection and quantification of plasmid‐mediated colistin resistance genes (*mcr*‐1 to *mcr*‐5) by real‐time PCR in bacterial and environmental samples. *Journal of Applied Microbiology* **129**, 1523-1529 (2020).

5 Poirel, L., Walsh, T. R., Cuvillier, V. & Nordmann, P. Multiplex PCR for detection of acquired carbapenemase genes. *Diagnostic microbiology and infectious disease* **70**, 119-123 (2011).

6 Ellington, M. J., Kistler, J., Livermore, D. M. & Woodford, N. Multiplex PCR for rapid detection of genes encoding acquired metallo-β-lactamases. *Journal of antimicrobial chemotherapy* **59**, 321-322 (2007).

7 Gomes, A. É. I. *et al.* Selection and validation of reference genes for gene expression studies in *Klebsiella pneumoniae* using Reverse Transcription Quantitative real-time PCR. *Scientific reports* **8**, 1-14 (2018).

8 Jayol, A., Nordmann, P., Brink, A. & Poirel, L. Heteroresistance to colistin in *Klebsiella pneumoniae* associated with alterations in the PhoPQ regulatory system. *Antimicrobial agents and chemotherapy* **59**, 2780-2784 (2015).

9 Srinivasan, R. *et al.* Use of 16S rRNA gene for identification of a broad range of clinically relevant bacterial pathogens. *PloS one* **10**, e0117617 (2015).

10 Compain, F. *et al.* Multiplex PCR for detection of seven virulence factors and K1/K2 capsular serotypes of *Klebsiella pneumoniae*. *Journal of clinical microbiology* **52**, 4377-4380 (2014).

11 Vuotto, C. *et al.* Biofilm formation and antibiotic resistance in *Klebsiella pneumoniae* urinary strains. *Journal of applied microbiology* **123**, 1003-1018 (2017).

**References**
